# Frailty: from concept to clinical practice in urology

**DOI:** 10.3389/fmed.2026.1907872

**Published:** 2026-08-26

**Authors:** Lazaros Tzelves, Stamatios Katsimperis, Catherine Meilak, Adrian Wagg, Bård Reiakvam Kittang, Stephen Baug, Elisabeth Grov Beisland, Rifat Burak Ergül, Christian Beisland, Bhaskar Somani, Patrick Juliebø-Jones

**Affiliations:** 1 Second Department of Urology, National and Kapodistrian University of Athens, Sismanogleio Hospital, Athens, Greece; 2 Department of Geriatric Medicine, East Kent Hospitals University NHS Foundation Trust, Canterbury, United Kingdom; 3 Division of Geriatric Medicine, Department of Medicine, Faculty of Medicine & Dentistry, College of Health Sciences, University of Alberta, Edmonton, Canada; 4 Department of Medicine, Haraldsplass Deaconess Hospital, Bergen, Norway; 5 Department of Clinical Science, University of Bergen, Bergen, Norway; 6 Department of Nursing Home Medicine, Municipality of Bergen, Bergen, Norway; 7 Department of Urology, Haraldsplass Deaconess Hospital, Bergen, Norway; 8 Faculty of Health and Social Sciences, Western Norway University of Applied Sciences, Bergen, Norway; 9 Department of Urology, Mus State Hospital, Mus, Türkiye; 10 Department of Urology, Haukeland University Hospital, Bergen, Norway; 11 Department of Urology, Southampton University Hospital, Southampton, United Kingdom

**Keywords:** comprehensive geriatric assessment, frailty, older adults, perioperative care, urology

## Abstract

**Introduction:**

Failty refers to a state of vulnerability to stressors caused by declines in physiological reserve across multiple systems and is associated with adverse health outcomes. Despite its established importance in medical specialties, frailty assessment remains inconsistently implemented in urology. The aim of this review was to provide an overview of the concept of frailty and summarise the current evidence regarding its relevance to urological practice.

**Methods:**

A narrative literature review was performed using PubMed/MEDLINE and Google Scholar. Articles relating to frailty, geriatric and perioperative care, surgical outcomes, and urology were identified and reviewed. Findings were synthesised to provide a clinical overview relevant to healthcare professionals working in urological settings.

**Results:**

Frailty is common among older adults and is increasingly recognised as a marker of vulnerability beyond chronological age alone. Multiple validated assessment tools are available, including the Clinical Frailty Scale and Frailty Index, although no single instrument has demonstrated clear superiority. Existing evidence suggests that frailty is associated with higher rates of postoperative complications, mortality, prolonged hospitalisation, and increased healthcare utilisation in urological patients. Studies further indicate that comprehensive geriatric assessment may identify potentially modifiable risk factors and support frailty-informed care pathways. While most published data relate to elective uro-oncological surgery, emerging evidence from acute urology suggests that integration of geriatric services may improve outcomes such as length of stay and hospital readmission.

**Conclusions:**

Frailty is highly relevant to contemporary urological practice and appears to be a stronger predictor of adverse outcomes than chronological age alone. Routine frailty assessment using simple validated tools may enhance risk stratification, perioperative planning, and shared decision-making. Wider implementation of frailty-informed care pathways and greater incorporation of frailty measures into urological research should be prioritised as the population ages.

## Introduction

Frailty refers to a condition of vulnerability to stressors induced by declines in reserve and function in multiple physiological systems, and is characterised by a diminished and slower response, leading to a higher likelihood of adverse outcomes and functional decline (1). As compensatory capacity diminishes, even seemingly minor stressors, such as a urinary tract infection, may precipitate disproportionately significant functional decline (2). Notably, impairment in the ability to perform activities of daily living independently is identified as a strong predictor of diminished quality of life (3). A useful analogy is that of a tower of stacked bricks: as physiological reserve declines, the removal of even a small “piece”, such as a minor stressor or intervention, may lead to collapse of the entire structure (4).

For urologists, frailty has particular clinical relevance. Urology manages one of the oldest patient populations across all surgical specialties, with many patients presenting with uro-oncological disease as well as benign conditions such as lower urinary tract symptoms, urinary stone disease, recurrent urinary tract infection, urinary incontinence, and benign prostatic obstruction (5). As life expectancy continues to increase, the number of older adults requiring urological assessment and intervention is expected to rise, making frailty assessment an increasingly important component of routine urological practice.

However, despite its important clinical relevance, recognition and implementation of frailty assessment and management strategies remain variable across healthcare disciplines. While well-established within medical specialties, systematic frailty assessment is less developed in surgical disciplines, with orthopaedics being recognised as one of the exceptions (6).

The comparatively limited emphasis on frailty within urology relative to other medical fields is paradoxical given the advanced age of many urological patients and the growing body of evidence demonstrating frailty as a strong predictor of postoperative morbidity and mortality across surgical discipline (7). Furthermore, an expanding body of literature demonstrates that frailty is associated with increased postoperative complications, prolonged length of stay, readmission, nursing home admission, mortality, and impaired functional recovery following major urological surgery (8, 9). Importantly, frailty assessment is not intended to deny treatment, but rather to facilitate risk stratification, shared decision-making, perioperative optimisation, comprehensive geriatric assessment (CGA) where appropriate, and targeted interventions such as exercise- and nutrition-based prehabilitation that may improve postoperative outcomes.

The aim of this literature review was twofold: to provide an overview of the concept of frailty and to summarize the current evidence on frailty in urology as a clinical primer for healthcare professionals working in urological settings (10). In addition, this review highlights the prevalence and impact of frailty across contemporary urological practice, summarises current evidence regarding perioperative optimisation and prehabilitation, and discusses how frailty assessment can inform surgical decision-making and improve patient-centred outcomes in both elective and emergency urology.

## Methods

A narrative literature review was undertaken to identify evidence relating to frailty and its relevance to contemporary urological practice. Electronic searches were performed using PubMed/MEDLINE and Google Scholar between May and August 2026. Search terms included combinations of *frailty*, *frailty index*, *Clinical Frailty Scale*, *comprehensive geriatric assessment*, *geriatric care*, *perioperative care*, *urology*, *uro-oncology*, *older adults*, and *surgery*, combined using Boolean operators (AND/OR). Original research articles, systematic reviews, meta-analyses, narrative reviews, and relevant clinical guidelines published in English were considered. No formal date restrictions were applied. Articles were selected based on their relevance to the concept of frailty, frailty assessment, perioperative medicine, and urological practice, with emphasis placed on landmark publications and contemporary evidence. Findings were synthesised narratively to provide a clinically focused overview for healthcare professionals working in urological settings.

### Frailty as a construct

While several models of frailty have been proposed, two predominate. The Fried frailty phenotype is based on physical function and incorporates five domains: unintentional weight loss (>10% of body weight), self-reported exhaustion, weakness as measured by reduced grip strength, low levels of physical activity, and slow walking speed. Patients meeting three or more of these criteria are considered frail, while those meeting one or two criteria are classified as pre-frail (11). This model conceptualises frailty as a distinct biological syndrome. Although the original model does not specify a particular order in terms of symptom onset, longitudinal studies suggest that low physical activity and fatigue often appear earlier in the trajectory, whereas unintentional weight loss is more commonly associated with later stages of frailty progression (12). Individuals meeting three or more criteria are classified as frail, those with one or two as pre-frail, and those with none as robust. Of note, the model does not account for cognitive or social frailty.

The other construct, the Frailty Index (FI), views frailty as an accumulation of deficits across multiple domains with frailty representing a state of continuum (13). The FI functions both as a conceptual model and as a measurement tool. The original model, incorporated up to 70 possible deficits, derived from the CGA expressed as a fraction of the number of deficits over the total number counted. The FI was primarily suited to research rather than routine clinical use but since its development, multiple adaptations, including shorter and modified versions, many based upon administrative datasets, have been produced to improve its implementation in different settings and disciplines (14, 15). Of note, FI is distinct from the Clinical Frailty Scale (CFS), often referred to as the Rockwood scale, which is a global clinical judgement tool that characterises frailty on a 1–9 scale (16). However, there is noted to be a high correlation between CFS score and the mathematically derived FI (Pearson 0.80, *p* < 0.01) (re(13)f). Indeed, despite their differences, the two models are best regarded as complementary rather than competing. In fact, when administered the many differing frailty indices and scales do identify different but overlapping cohorts of people.

### Transitions across frailty states

Frailty is a dynamic rather than a fixed process. Although the overall trajectory tends to be one of decline, movement between states can occur in both directions. In a longitudinal meta-analysis by Kojima et al., 54.5% of individuals who were frail at baseline remained frail at follow-up, while 40.3% improved to a pre-frail state and only 3.3% recovered to robust/normal state (17).

Improvements are more commonly observed in younger individuals, females and those from higher socioeconomic groups, whereas conditions such as dementia or cancer substantially reduce the likelihood of improvement (1, 18, 19). In a population based study., Gill et al., found that each hospitalisation substantially undermines recovery potential, reducing the likelihood of transitioning to a less frail state by about half (20).

### Prevalence of frailty

In a meta-analysis including patients from 62 countries, the pooled prevalence of frailty ranged from 12%–24%, reflecting differences between frailty models, while pre-frailty was highly prevalent across populations at rates of 46%–49% (21). In another meta-analysis including nearly half a million hospital inpatients aged ≥65 years, the prevalence of frailty was 47% (22). A higher proportion of frail patients has been documented among certain established disease groups such as chronic obstructive pulmonary disease (COPD) and end stage renal disease (23). The recent SNAP-3 study, a prospective multicentre UK cohort study, found that approximately one in five inpatient surgical patients lives with frailty. Frailty was associated with poorer postoperative outcomes and appeared to be a stronger predictor of adverse outcomes than multimorbidity (24).

While frailty should not be considered synonymous with old age, its prevalence is greater among older adults (21). This is largely attributable to the loss of physiological reserve associated with ageing, including factors such as sarcopenia and hormonal dysregulation. As such, frailty is often referred to as a geriatric syndrome (25). However, given that at least half of individuals over 85 are not frail suggests that frailty is not solely a consequence of ageing, but reflects multiple interactingbiological processes (2). As such, frailty may be considered a marker of physiological rather than chronological age; however, it is more accurately defined by impaired physiological reserve and resilience to stressors than by physiological age alone.

### Economic impact of frailty

Frailty is a key driver for healthcare system costs. In a longitudinal study of more than 2 million patients in England, Fogg et al. reported that annual healthcare costs doubled in mild frailty, tripled in moderate frailty, and quadrupled in severe frailty (26). The main contributors to the burden were unplanned hospital admissions, medication use, the number of inpatient bed days and more frequent contact with primary care services. in a meta-analysis of 51 studies Pradana et al. reported that adults aged 60 and over living with frailty incur substantially higher healthcare costs, with annual expenditures exceeding those of pre-frail and robust individuals by US $3,286 and US $4,653, respectively (27). The implications are far reaching and affect multiple aspects of healthcare, often in ways that are not immediately obvious. For example, a study of ambulance services from the United Kingdom (UK) found that median on-scene time was significantly longer when the patient was frail, highlighting how frailty can increase complexity and resource use even before hospital care begins (28). Canadian data suggest that frailty is associated with markedly higher healthcare system use and, in Ontario home-care recipients, roughly C$11,000–C$12,000 (2019 $) higher publicly funded healthcare costs per person per year compared with robust older adults (29).

### Risk factors for frailty

Risk factors for frailty can be categorised across biological, clinical, and social domains, recognizing the complex interaction between physical, social and cognitive frailty (30). Physiological factors include immune system dysfunction, malnutrition, and anemia. Comorbidities such as cardiovascular disease, stroke, diabetes and smoking are well-established risk factors for frailty development (31). The increased prevalence of cognitive impairment in later life is a significant contributor (32). Social determinants of health, including lower socioeconomic status, have also been consistently associated with incident frailty. Increasing age and female sex are strong demographic risk factors; these represent key non-modifiable determinants (33).

### Use of validated tools to assess frailty

Health care professionals often largely rely on intuition and clinical judgement to identify frailty, with systematic approaches often lacking (34). The use of clinical judgment alone, representing a subjective assessment, frequently underestimates the actual burden of frailty relative to structured evaluations (35).

Tools for assessing frailty can be categorised into three broad groups: (1) clinician-based assessments (e.g., CFS), (2) patient self-assessment instruments (e.g., PRISMA-7), and (3) electronically derived measures using routinely collected electronic health record data (e.g., electronic Frailty Index (eFI)).

There is currently limited evidence to support the objective superiority of one tool over another (36). In the urological setting, a prospective study of older adults with pelvic floor disorders demonstrated that the 9-point CFS showed good agreement with the Fried Frailty Phenotype, supporting its use as a rapid screening tool in routine preoperative assessment (37). Different frailty assessment methods have distinct strengths and limitations. However, in terms of usability in everyday clinical practice, a multicenter study found the CFS to be preferred by nurses in the emergency care setting (38). The CFS is a simple, rapid tool that does not require equipment and can be used by clinicians without specialist geriatric training. It is intended to reflect an individual's baseline state two weeks prior to the assessment (e.g., hospital admission). This reliance on recent functional history means that collateral information is often necessary, and retrospective scoring based solely on medical records may be inaccurate.

### Comprehensive geriatric assessment

Once frailty has been identified, the next step may be to arrange a comprehensive geriatric assessment (CGA), which enables the identification of potentially treatable conditions and the development of a targeted management plan (39). It also facilitates the development of a coordinated care and support plan that incorporates the patient's individual goals and priorities. CGA spans multiple domains, including medical, functional, cognitive, and psychological factors, and is delivered by a multidisciplinary team.

Although awareness of CGA is important, urologists are not responsible for its delivery (40). Rather, it is led by geriatricians CGA is an evidence informed model and carried out by a multidisciplinary team that includes nurses, allied health professionals (such as physiotherapists, occupational therapists, speech and language therapists, and dietitians), as well as pharmacists and social workers. Within this framework, urologists contribute by identifying suitable candidates, initiating referral pathways where available, and integrating CGA outputs into shared decision-making and perioperative management. CGA is an evidence informed framework which, when operationalised, improves clinical outcomes for older adults (41).

### Prevalence of frailty in urology patients

No studies quantifying the burden of frailty across all urology patients were retrieved, although age-profile data suggests that the burden might be high. For example, Lee et al., reported that over a 3 year period at a university hospital in South Korea, 71% of all urology inpatients were over 60 years old (42). Similarly, in a national study of the 120 urological departments in Poland, 67% were ≥60 years (5).

Although data describing frailty across all urological conditions remain limited, studies in major uro-oncology suggest that frailty is common. In a study of 68 patients undergoing major uro-oncology surgery at a unit in Poland, 39.7% were formally classified as frail using CGA (43). Similarly, a retrospective analysis of the National Inpatient Sample (NIS) database found that 16% of patients undergoing radical nephroureterectomy were classified as frail using the Johns Hopkins Frailty Indicator (44). Similarly, another NIS study reported that 24.3% undergoing radical cystectomy were classified as frail using the same frailty measure (45). Collectively, these findings suggest that frailty is common among patients undergoing major urological surgery, particularly in uro-oncology, reinforcing the need for routine frailty assessment within this population.

Although having lower urinary tract symptoms (LUTS) is not considered a risk factor for frailty, a cross-sectional study by Soma et al. reported higher frailty burden among patients presenting with LUTS. This association suggests that LUTS can act as a practical clinical prompt for frailty screening in urology settings (46), a finding that has also been supported by other studies (47).

### Frailty in the elective urology setting

In both urology and other surgical specialties, research on frailty, encompassing both observational and interventional studies, has predominantly been conducted in the elective setting (Tables 1, 2). The literature is consistent in demonstrating that frailty is associated with higher rates of major complications at 30 and 90 days, as well as increased 30-day mortality, particularly in the context of major uro-oncological surgery (48). A systematic review of patients undergoing radical cystectomy found that frailty and pre-frailty were associated with higher rates of major postoperative complications, prolonged length of stay, increased healthcare costs, non-home discharge and early mortality compared with non-frail patients (49). This association extends across other uro-oncological procedures. In patients undergoing radical nephroureterectomy (50), increasing frailty was associated with postoperative acute kidney injury, thromboembolic events, greater blood loss, prolonged hospitalisation, higher readmission rates, and worse progression-free and overall survival.

**Table 1:** Key studies on frailty in uro-oncological surgery.

Year	Author	Study type	Procedure/condition	Key findings
2021	Campi et al	Systematic review	Renal cancer surgery	Frailty was consistently associated with worse perioperative outcomes (complications, readmissions, longer hospitalisation) and poorer survival outcomes following surgery or ablation for RCC.
2022	Rosiello et al	Retrospective analysis of the National Inpatient Sample	Partial nephrectomy	Frailty was independently associated with worse perioperative outcomes and increased healthcare resource utilization after partial nephrectomy.
2021	Gontero et al	Prospective multicentre cohort study	Partial nephrectomy	Patients with limited life expectancy had higher overall complication rates after partial nephrectomy, but major complications and functional outcomes were comparable to those with longer life expectancy.
2020	Rosiello et al	Retrospective analysis of the National Inpatient Sample	Radical prostatectomy	Frailty was independently associated with increased overall and major postoperative complications, longer length of stay, higher hospital costs, and greater likelihood of non-home discharge following radical prostatectomy.
2025	Stankovic et al	Retrospective cohort study	Radical prostatectomy	Frailty independently predicted poorer early continence recovery.
2020	Palumbo	Retrospective analysis of the National Inpatient Sample	Radical cystectomy	Frailty was the strongest predictor of early adverse outcomes after radical cystectomy, independently predicting complications, failure to rescue, in-hospital mortality, longer hospitalization, and higher costs.
2023	Yu et al	Retrospective cohort study	Radical cystectomy	Higher preoperative frailty was independently associated with increased 1-year all-cause and cancer-specific mortality after radical cystectomy.
2020	Vlies et al	Prospective single-centre cohort study	Radical cystectomy	Frailty independently predicted severe postoperative complications and 1-year mortality after radical cystectomy and improved risk prediction beyond ASA score and comorbidity indices.
2019	Burg et al	Prospective single-centre cohort study	Radical cystectomy	Fried frailty status predicted high-grade postoperative complications after radical cystectomy, while individual frailty components predicted overall complication risk.

**Table 2:** Key studies on frailty in setting of benign urology.

Year	Author	Study type	Procedure/condition	Key findings
2019	Medendorp et al	Retrospective cohort study (ACS-NSQIP database)	Male stress incnontinence surgery	Frailty was associated with increased major complications and a higher likelihood of artificial urinary sphincter removal, outperforming age as a predictor of adverse outcomes.
2022	Van Kuiken et al	Retrospective population-based cohort study	Female stress incontinence surgery	Increasing frailty was associated with higher rates of 30-day postoperative complications, 1-year mortality, and repeat procedures for persistent incontinence or obstructed voiding after sling surgery.
2023	Ren et al	Prospective longitudinal cohort study	TURP	Frailty independently predicted poorer improvement in LUTS and quality of life following TURP for BPH.
2023	Elsaqa et al	Retrospective single-centre cohort study	HoLEP	Frailty predicted higher early postoperative morbidity and readmission after HoLEP but did not adversely affect longer-term functional outcomes or continence
2022	Labban et al	Retrospective registry-based cohort study (ACS-NSQIP)	BPO surgery	Frailty independently predicted adverse 30-day outcomes after endoscopic BPO surgery, while laser-based procedures were associated with lower complication rates than TURP.
2023	Uwumiro et al	Retrospective analysis of the National Inpatient Sample	Kidney stone surgery	Frailty predicted higher perioperative morbidity, healthcare costs, and non-home discharge in patients undergoing treatment for urolithiasis, but not in-hospital mortality.
2024	Moura et al	Retrospective single-centre cohort study	Adult-acquired buried penis surgery	Frailty was an independent predictor of postoperative complications and poorer patient-reported outcomes following buried penis surgery, outperforming BMI and ASA status for risk stratification.

Frailty has also been shown to influence outcomes following diagnostic procedures. In a population-based study of 193,490 men undergoing prostate needle biopsy, frailty was independently associated with an increased risk of overall complications, emergency department attendance, hospital readmission and outpatient clinic visits within 30 days of biopsy, highlighting that the clinical impact of frailty extends beyond major surgery to diagnostic interventions (51).

Implementation of perioperative clinics that facilitate CGA can allow for identification of modifiable risk factors that can be optimised prior to surgery. Emerging evidence from bladder cancer also demonstrates that CGA has a direct impact on clinical decision-making. In a prospective cohort of older patients with high-risk non-muscle-invasive and muscle-invasive bladder cancer, CGA influenced multidisciplinary treatment selection in 19% of patients, most commonly through treatment de-escalation, while also prompting medication review, prehabilitation advice, postoperative monitoring and other patient-specific optimisation strategies (52).

In a large US study of more than 50,000 patients (across a wide range of specialities), implementation of routine preoperative frailty assessment was associated with an 18% reduction in the odds of 1-year postoperative mortality (53). The observed effect was considered multifactorial, likely reflecting improved documentation of frailty-informed shared decision-making, better alignment of surgical intervention with patient goals and physiological reserve, perioperative optimisation, and, in some cases, selection of non-operative management.

Although most evidence relates to uro-oncology, frailty is increasingly recognised across the broader spectrum of urological practice.

For example, analyses of two nationally representative population cohorts from China and the United States demonstrated that increasing frailty burden was independently associated with symptomatic benign prostatic hyperplasia/lower urinary tract symptoms, supporting the relevance of frailty beyond oncological disease (54). Frailty has also been shown to influence outcomes following surgery for stress urinary incontinence. In a Medicare cohort of more than 7,000 men undergoing artificial urinary sphincter or male sling surgery, frailty was independently associated with an increased risk of 30-day postoperative complications and subsequent device revision or removal, irrespective of age and comorbidity (55).

Older adults presenting with benign prostatic obstruction, recurrent urinary tract infection, urinary stone disease, urinary incontinence, or chronic urinary retention frequently have multimorbidity and reduced physiological reserve. While frailty has been described in these populations, evidence evaluating frailty-guided interventions, multidisciplinary care pathways, and optimisation strategies remains limited.

### Frailty in the acute urology setting

Older adults account for a substantial proportion of emergency urological admissions, with frailty frequently complicating the management of conditions such as obstructive uropathy with sepsis, acute urinary retention, severe haematuria, infected urinary stones and complications of urinary diversion. Despite this, relatively little research has specifically evaluated frailty in emergency urology. In a cohort of patients undergoing emergency nephrostomy placement or exchange, 32% were classified as frail using the Clinical Frailty Scale, demonstrating the high burden of frailty among patients requiring urgent upper urinary tract decompression (56). Recognition of frailty in the emergency setting may therefore inform perioperative risk assessment, goals-of-care discussions, discharge planning and early involvement of multidisciplinary teams, even when opportunities for preoperative optimisation are limited. Braude et al., reported on the impact of embedding a geriatric liaison service on an inpatient urology ward. This intervention resulted in a significant reduction in length of stay, fewer operations cancelled and lower rate of hospital readmissions (57). Evans et al. described a service model in which geriatric medicine specialists contributed on a bi-weekly basis to twice-weekly multidisciplinary team (MDT) meetings with geriatric liaison and urology teams, leading to a reduction in hospital length of stay (58). These findings suggest that integrating geriatric expertise into acute urological care may improve outcomes, although access to specialist geriatric services remains variable between institutions (59).

Although it may seem counterintuitive, hospital admission is often not conducive to the health of patients living with frailty; prolonged bed rest, immobility, disrupted sleep routines, and polypharmacy accelerate physiological decline. Up to 30% of hospitalized older adults loses independence in at least one activity of daily living as a result of acute hospitalisation (60). There is a measurable loss of lean muscle mass related to hospitalization in older adults (61). Although admission may feel protective, a timely, well-planned discharge to the usual place of residence may be more beneficial for selected patients.

Many hospitals have an acute frailty service that can be contacted as well as even a dedicated inpatient ward (57). Where implemented, acute frailty services have demonstrated significant benefits, including reduced time spent in the emergency department, lower rates of nursing home admission, improved quality of life, and reduced functional decline at 30-day follow-up (62). Although the available evidence is encouraging, prospective multicentre studies are needed to determine whether frailty screening and dedicated geriatric-urology pathways improve surgical outcomes, functional recovery, discharge destination and healthcare utilisation in emergency urological patients.

### Psychological and social considerations in frailty

While the Fried phenotypical model focuses on physical impairments, some modified versions of the cumulative deficits model psychological elements such as low mood and cognitive decline. The impact that elements such as loneliness, grief and economic hardship can have, implies that *social frailty* is an area that is being increasingly studied, resulting in the development of Social Frailty Indices (63). As chronological age alone should not be used as an absolute criterion when considering operative intervention, frailty should similarly not be viewed as an exclusionary factor. Rather, assessment of frailty should inform discussions regarding anticipated risks, outcomes, and patient expectations, and should be incorporated into shared decision-making. Importantly, some older adults perceive the term frailty as stigmatising, which may influence how it is discussed and understood in clinical practice. Beyond physical outcomes, frailty has also been associated with increased depressive symptoms following radical prostatectomy, with sleep disturbance partially mediating this relationship (64).

Rather, it should form the basis for shared decision-making, allowing patients, families, and clinicians to consider which treatments are most likely to be truly beneficial. The multidimensional connotations of psychological frailty and the overlap between multiple domains of frailty require more attention in future discussions of a consistent definition of psychological frailty.

### Implications for practice

Frailty assessment should be routinely incorporated into the evaluation of older adults being considered for operative intervention. The Clinical Frailty Scale (CFS) represents a simple, practical, and easily implemented screening tool that can be used in both acute and elective settings. Identification of frailty should trigger referral for specialist geriatric assessment and the implementation of frailty-informed care pathways aimed at improving patient outcomes. In the inpatient setting, this may include the delivery of older adult-friendly models of care and multidisciplinary management, while in the outpatient setting opportunities for optimisation should be pursued. Where operative intervention remains appropriate and sufficient time is available, targeted prehabilitation strategies, including exercise and nutritional interventions, should be considered to improve physiological reserve and perioperative outcomes. Importantly, frailty assessment should be used to facilitate risk stratification, optimisation, and shared decision-making rather than to exclude patients from potentially beneficial treatment.

### Future research on frailty in urology

In urology, frailty is not yet routinely captured as a baseline parameter in large-scale observational studies in urology, in contrast to established perioperative measures such as the ECOG and American Society of Anesthesiologists (ASA) physical status classification. Similarly, outcome assessment continues to focus primarily on traditional endpoints such as survival, rather than frailty trajectories or functional status (65). This evidence gap is also reflected in contemporary urological guidelines, where frailty assessment and age-related management strategies remain inconsistently integrated across many subspecialties, highlighting the need for broader incorporation of frailty-informed assessment and patient-centred outcomes into future guideline development (66).

Although frailty has been investigated beyond uro-oncology, particularly in benign, functional, and emergency urology, much of the available evidence remains observational. Future studies should address this by evaluating whether frailty-informed interventions, including CGA, multidisciplinary perioperative care, prehabilitation, medication optimisation, and nutritional support, improve perioperative, functional, and patient-reported outcomes across these settings. Likewise, greater emphasis should be placed on outcomes that matter to older adults, including functional recovery, maintenance of independence, quality of life, and treatment burden, rather than relying solely on traditional surgical endpoints such as complications and mortality.

Likewise, there has been little work on identifying outcomes that are of importance to older adults living with frailty. As research attention increasingly turns to what has been termed the “forgotten majority” of older adults with complex needs, greater emphasis is needed on implementing interventions to modify outcomes, given variable findings from studies to date (67, 68). For example, whether targeted interventions like prehabilitation and medication consolidation have measurable effect over time (69). This shift is expected to be complemented by broader advances in the field, including from a basic sciences perspective, the development of candidate frailty biomarkers.

This review has several limitations. As a narrative review, it was not designed as a systematic review and therefore did not include a formal assessment of study quality or risk of bias. The available literature is heterogeneous with respect to study design, patient populations, frailty assessment tools, and reported outcomes, limiting direct comparison between studies. In addition, much of the current evidence is derived from observational studies, and high-quality prospective interventional data remain limited. Furthermore, only English-language publications were included, which may have excluded relevant evidence published in other languages. Nevertheless, the review aimed to provide a clinically focused overview of the current evidence and highlight areas requiring further research.

## Conclusion

Frailty is an increasingly important consideration in urological practice as the population ages. Although most evidence derives from uro-oncology, frailty is relevant across the broader spectrum of urology. Routine frailty assessment using simple, validated tools can improve risk stratification, shared decision-making, and multidisciplinary care. Future research should focus on determining whether frailty-informed interventions improve patient-centred and perioperative outcomes across all areas of urological practice.
